# Improved performance of machine learning models in predicting length of stay, discharge disposition, and inpatient mortality after total knee arthroplasty using patient-specific variables

**DOI:** 10.1186/s42836-023-00187-2

**Published:** 2023-07-02

**Authors:** Leo Zalikha, Tannor Court, Fong Nham, Mouhanad M. El-Othmani, Roshan P. Shah

**Affiliations:** 1 Department of Orthopaedic Surgery and Sports Medicine, Detroit Medical Center, Detroit, MI 48201 USA; 2 Department of Orthopaedic Surgery, Columbia University Medical Center, New York, NY 10032 USA

**Keywords:** Machine learning, Total knee arthroplasty, Artificial intelligence, Postoperative outcomes, Length of stay

## Abstract

**Background:**

This study aimed to compare the performance of ten predictive models using different machine learning (ML) algorithms and compare the performance of models developed using patient-specific vs. situational variables in predicting select outcomes after primary TKA.

**Methods:**

Data from 2016 to 2017 from the National Inpatient Sample were used to identify 305,577 discharges undergoing primary TKA, which were included in the training, testing, and validation of 10 ML models. 15 predictive variables consisting of 8 patient-specific and 7 situational variables were utilized to predict length of stay (LOS), discharge disposition, and mortality. Using the best performing algorithms, models trained using either 8 patient-specific and 7 situational variables were then developed and compared.

**Results:**

For models developed using all 15 variables, Linear Support Vector Machine (LSVM) was the most responsive model for predicting LOS. LSVM and XGT Boost Tree were equivalently most responsive for predicting discharge disposition. LSVM and XGT Boost Linear were equivalently most responsive for predicting mortality. Decision List, CHAID, and LSVM were the most reliable models for predicting LOS and discharge disposition, while XGT Boost Tree, Decision List, LSVM, and CHAID were most reliable for mortality. Models developed using the 8 patient-specific variables outperformed those developed using the 7 situational variables, with few exceptions.

**Conclusion:**

This study revealed that performance of different models varied, ranging from poor to excellent, and demonstrated that models developed using patient-specific variables were typically better predictive of quality metrics after TKA than those developed employing situational variables.

**Level of Evidence:**

III.

**Supplementary Information:**

The online version contains supplementary material available at 10.1186/s42836-023-00187-2.

## Introduction

Total knee arthroplasty (TKA) is a safe and effective treatment for end-stage osteoarthritis and is among the most common surgical procedures performed in the USA. National projections anticipate a substantial increase in TKA utilization and its associated economic burden well into the foreseeable future [1]. As healthcare systems shift toward an increasing focus on value and patient satisfaction, there has been increased emphasis placed on risk stratification, perioperative optimization, and improving value in TKA care delivery [2–4]. As such, considerable effort has been put forth to develop models to predict clinical outcomes after TKA [5]. More recently, artificial intelligence and machine learning have been heavily explored as potential tools to improve the predictive capacity of these models [6–8].

Machine learning (ML) is a subset of artificial intelligence (AI) that automates analytical model building by employing algorithms to progressively “learn” and improve from data [9, 10]. As technological shifts in the healthcare system have allowed for the accumulation and organization of large amounts of data, ML has shown immense promise for numerous applications within healthcare system. Recent studies within the orthopedic literature have applied ML to develop models to predict mortality, readmission rates, complication rates, length of stay, and patient-reported postoperative outcomes [8, 11–15]. Such predictive models have numerous potential benefits, including identifying patients at risk for worse outcomes, which allows for improved patient selection, targeted perioperative optimization, and stratification for risk-based alternative payment models. While these promising studies demonstrate the potential of ML to predict outcomes and improve value within orthopedics, they typically are limited in their choice of training variables and often employ a single elementary algorithm, without justification for the selection of either algorithm or variables. As a whole, there remains a critical need to develop and comparatively analyze the predictive capacity of various ML algorithms and to identify and select the relevant input variables used to train these models.

In that context, the purpose of this study was to comparatively evaluate the performance of ten different machine learning models in predicting LOS, mortality, and discharge disposition following TKA and to compare the performance of the best performing models developed with patient-specific vs. situational variables.

## Methods

### Data source and study sample

The National Inpatient Sample, a public and expansive database containing data of more than 7 million hospital stays in the US for the years 2016 and 2017, was utilized for this retrospective analysis and ML model development. Given the use of the International Classification of Disease, Tenth Revision (ICD-10) coding system in the database during the study period, the ICD-10-Procedure Coding System (ICD-10-PCS) for TKA was utilized to identify the study population (Additional file 1). Patients undergoing a conversion or revision TKA, younger than 18 years of age, or missing age information were excluded from the study population. This strategy resulted in a total of 305,577 discharges that were included in the current study.

### Predictive and outcome variables selection

All available variables in the NIS database were considered and assessed for inclusion in this study. For the initial step of the study, 15 predictive variables were included in building and assessing ten different ML models, and subsequently divided, in the second step of the study, into 8 patient-specific (including Age, Sex, Race, Total number of diagnoses, All Patient Refined Diagnosis Related Groups (APRDRG) Severity of illness, APRDRG Mortality risk, Income zip quartile, Primary payer) and 7 situational variables (including Patient Location, Month of the procedure, Hospital Division, Hospital Region, Hospital Teaching status, Hospital Bed size, and Hospital Control). These features were manually selected by the authors by screening from all available variables in the NIS database. The analysis outcome variables were in-hospital mortality (binary yes/no outcome), discharge disposition (home vs. facility), and length of stay (≤ 2 vs. > 2) among primary TKA recipients. The determination of the LOS cutoff level was guided by analysis of the average LOS for the entire cohort, and subsequently utilizing the closest lower integral number to create the binary outcomes. Patient discharge destination was coded as either home (discharge to home or home health care) or facility (all other dispositions to a facility, such as skilled nursing facilities or inpatient rehabilitation centers). Patient datasets missing information on these variables were removed from the study sample.

### Data handling and machine learning models development

SPSS Modeler (IBM, Armonk, NY, USA), a data mining and predictive analytics software, was utilized to develop the models based on commonly used ML techniques. The algorithmic methods implemented included Random Forest (RF), Neural Network (NN), Extreme Gradient Boost Tree (XGBoost Tree), Extreme Gradient Boost Linear (XGBoost Linear), Linear Support Vector Machine (LSVM), Chi square Automatic Interaction Detector (CHAID), Decision lists, Linear Discriminant Analysis (Discriminant), Logistic Regression, and Bayesian Networks. These methods were selected as they are well-studied, commonly used ML methods in medical literature and are distinct in their pattern recognition methods (Table 1) [8–10, 12].

**Table 1:** Description of machine learning models

Machine Learning Models	Description
Random Forest (RF)	Qualitative algorithm using individual decision trees to generate a collective prediction. The strengths of this model are based on randomness utilizing methods such as bootstrapping, creating individual data sets through sampling, and bootstrap aggregating, otherwise known as bagging to shuffle individual variables each tree is trained. The algorithm works in a voting matter, so that the collective decision is supported by the number of individual trees that cast a vote
Neural Network (NN)	Network based on the working layers of neurons programmed to interpret data based on the channels and their corresponding weight in the forward propagation of decision making. Backpropagation trains the neurons by comparing the output with the correct output to generate the appropriate weight of each channel
Extreme Gradient Boost Tree (XGBoost Tree)	Expands on existing tree algorithms by further subtraining each tree in smaller subsets of data. The integration of small batch training strengthens an individual tree while the gradient boosting process uses the collective output from the trees. Gradient boosting builds upon sequential loss function to build the next generation of trees. This method occurs until the boosted ensemble can no longer improve upon the previous generation
Extreme Gradient Boost Linear (XGBoost Linear)	Similar to XGBoost tree, however, its utility is in features with less data-sets or low noise. The algorithm acts in a linear solution model with gradient boosting acting to build on the next rule until a rule can no longer improve upon the next generation. The speed is generally faster than that of XGBoost Tree, but accuracy is decreased if noise is high
Linear Support Vector Machine (LSVM)	Classifies a dataset using a regression algorithm with a small learning datasets. The model aims to divide the dataset into two classes. Each data point represents a distinct point in the Nth dimension of the hyperplane. LSVM maximizes the distances between the data points to determine the margin and to predict outcomes
Chi square Automatic Interaction Detector (CHAID)	Model based on the statistical differences between parent and child nodes given qualitative descriptors. The development requires large datasets to determine how to best identify patterns to generate accurate predictions
Decision lists	Boolean function model based on “if–then-else” statements with all subsets having either a true or false functional value, which is also known as an ordered rule set. Rules in this form are usually learned with a covering algorithm, learning one rule at a time The rules of this subset are tried in order unless no rule is induced, which pushes a default rule to be invoked
Linear Discriminant Analysis (Discriminant)	Calculate summary statistics of data by means and standard deviations. Using a training data source, new predictions are made when data are added and class labels are given based on each input feature. This machine learning method assumes input variables are normally distributed and therefore have the same overall variance
Logistic Regression	Similar to other linear regression models, but instead of solving for regression it acts to solve for classification. The input data sources can give a binary discrete value probability based on the independent variables of a given set. The benefit of logistic regression is its ability to classify observations and determine the most efficient observation group for classification, which can then be used to identify the probabilities of new data sets to fit into that classification
Bayesian Networks	Probabilistic graphical model of machine learning. They act to use a data source to identify probabilities for predictions, anomaly detections, and times predictions of an inputted data source. The data are computed into nodes which represent the variables that are linked to one another indicating their influence on one another. These links are a part of the structural learning and are identified automatically from the data. The data source can then be represented in graphical depictions called Asia networks making their data easy to understand following calculation

For each technique and for each outcome-variable, a new algorithm was developed. The overall data set was split using random sampling into three separate groups: a training, testing, and validation cohort. A total of 80% of the data were used to train-test the models, while the remaining 20% was employed to validate the model parameters. The training–testing subset was subsequently divided into 80% training and 20% testing, yielding a final distribution of 64% for training, 16% for testing, and 20% for model validation. In-between those phases, there were no leaks between the data sets, as mutually exclusive sets were used to train, test, and then validate each predictive algorithm.

When predicting outcomes with a low incidence rate, there exists a bias within the model, leading to an inaccurate imbalance in predictive capacity biased against the minority outcome [16]. As such, and to avoid such implications, when imbalanced outcome frequencies were encountered, the Synthetic Minority Oversampling Technique (SMOTE) was deployed to resample the training set to avoid any implications on the training of the ML classification [17, 18]. Despite the validation of SMOTE, as a measure to successfully minimize the impact of the bias, the classifier’s predictive ability in minority outcomes is improved, however, it remains imperfect.

### Statistical analysis

The comparative analysis of the different ML models consisted of assessment of responsiveness and reliability of the predictions for all models. Responsiveness is a measure of successful prediction of variable outcomes and was quantified with area under the curve (AUC) for the receiver operating characteristic (ROC) curve. AUCROC measurements were generated by assessing true positive rates vs. false positive rates under the training, testing, and validation phases of each model. For this study, responsiveness was considered as excellent for AUCROC was 0.90–1.00, good for 0.80–0.90, fair for 0.70–0.80, poor for 0.60–0.70, and fail for 0.50–0.60. Reliability of the ML models was measured by the overall performance accuracy quantified by the percentage of correct predictions achieved by the model.

All ten ML models were trained, tested, and validated to assess responsiveness and reliability. The first step of the study aimed at analyzing and comparing the predictive performance of these ML models in identifying the outcome variables after primary TKA: in-hospital mortality, discharge disposition, and LOS. The validation phase utilizing 20% of the sample was considered as the main assessment metric and quantified with responsiveness and reliability. Once the development and comparative assessment of the different ML models were completed, the three algorithmic methodologies with the highest accuracy for each outcome variable were identified. The second step of the study consisted of developing and comparing the predictive performance of the top three ML methodologies for the same set of outcome measures while using patient-specific and situational predictive variables. All statistical analyses were performed with SPSS Modeler version 18.2.2 (IBM, Armonk, NY, USA).

## Results

This study included a total of 305,577 discharges that underwent primary TKA with an average age of 66.51 years. Descriptive statistics for the distributions of the aforementioned predictive variables are included in Table 2. The study population had an average of 0.1% mortality during hospitalization, a home discharge rate of 79.6%, and an LOS of 2.41 days.

**Table 2:** Demographic variables of the study population

	***n* = 305,577**
**Age of Patient in Years: Mean (Mean Standard Error)**	66.51 (0.017)
**Biological Sex of Patient**	
Male	117,406 (38.4%)
Female	188,068 (61.6%)
**Primary Payor**	
Medicare	174,756 (57.2%)
Medicaid	13,334 (4.4%)
Private insurance	106,410 (34.8%)
Others	11,077 (3.6%)
**Race of Patient**	
White	237,015 (77.6%)
African American	23,930 (7.8%)
Hispanic	17,729 (5.8%
Asian or Pacific Islander	4,484 (1.5%)
Native American	1,243 (0.4%)
Other or Unknown	21,176 (6.92%)
**Median household income national quartile for patient ZIP Code**	
0–25th percentile	67,060 (21.9%)
26th to 50th percentile (median)	80,117 (26.2%)
51st to 75th percentile	81,480 (26.7%)
76th to 100th percentile	72,468 (23.7%)
Unknown	4,452 (1.5%)
**Bedsize of Hospital**	
Small	91,630 (30%)
Medium	87,561 (28.7%)
Large	126,386 (41.4%)
**Location/Teaching Status**	
Rural	31,225 (10.2%)
Urban Non-teaching	88,872 (29.1%)
Urban Teaching	185,480 (60.7%)
**Region of hospital**	
Northeast	53,637 (17.6%)
Midwest	81,590 (26.7%)
South	109,736 (35.9%)
West	60,614 (19.8%)
**Control/ownership of hospital (STRATA)**	
Government, non-federal	25,371 (8.3%)
Private, not-for-profit	229,407 (75.1%)
Private, investor-owned	50,799 (16.6%)
**Census Division of hospital**	
New England	15,200 (5%)
Middle Atlantic	38,437 (12.6%)
East North Central	54,530 (17.8%)
West North Central	27,060 (8.9%)
South Atlantic	57,054 (18.7%)
East South Central	20,712 (6.8%)
West South Central	31,970 (10.5%)
Mountain	23,494 (7.7%)
Pacific	37,120 (12.1%)
**Patient Location: NCHS Urban–Rural Code**	
Central counties of metro areas of ≥1 million population	68,832 (22.5%)
Fringe counties of metro areas of ≥1 million population	77,277 (25.3%)
Counties in metro areas of 250,000–999,999 population	67,499 (22.1%)
Counties in metro areas of 50,000–249,999 population	32,350 (10.6%)
Micropolitan counties	33,621 (11%)
Not metropolitan or micropolitan counties	25,702 (8.4%)
Unknown	296 (0.1%)
**APRDRG Risk Mortality**	
1- Minor likelihood of dying	252,204 (82.53%)
2- Moderate likelihood of dying	45,567 (14.91%)
3- Major likelihood of dying	6,529 (2.14%)
4- Extreme likelihood of dying	1,275 (042%)
**APRDRG Severity**	
1- Minor loss of function (includes cases with no comorbidity or complications)	156,092 (51.08%)
2- Moderate loss of function	134,776 (44.11%)
3- Major loss of function	13,748 (4.5%)
4- Extreme loss of function	959 (0.31%)
**Number of Diagnosis (Mean Standard Error)**	8.645 (0.009)
**Month of Procedure**	
January	26,732 (8.75%)
February	25,452 (8.33%)
March	25,874 (8.47%)
April	23,742 (7.77%)
May	25,437 (8.32%)
June	26,219 (8.58%)
July	21,861 (7.15%)
August	25,585 (8.37%)
September	23,868 (7.81%)
October	28,356 (9.28%)
November	27,730 (9.07%)
December	24,657 (8.07%)
**Died during hospitalization**	87 (0.1%)
**Disposition of patients**	
Discharged to Home	109,511 (35.8%)
Transfer to Short-term Hospital	736 (0.2%)
Transfer to Facility	60,768 (19.9%)
Home Health Care (HHC)	133,989 (43.8%)
Against Medical Advice (AMA) and Unknown	486 (0.2%)
**Length of Stay (Mean Standard Error)**	2.41 (0.003)

**Fig. 1:**
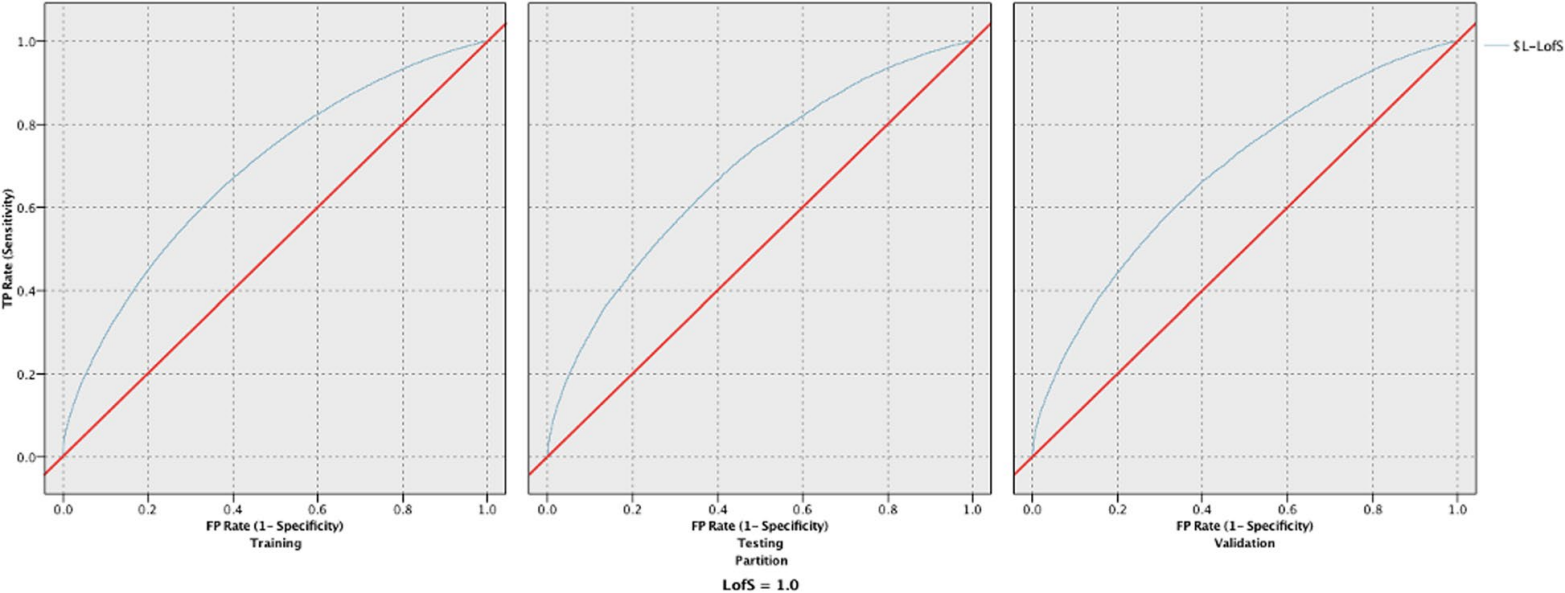
ROC curves for the training, testing, and validation phases for the LSVM model predicting LOS

**Fig. 2:**
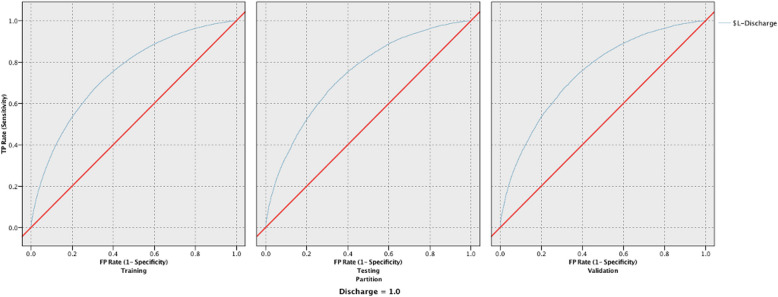
ROC curves for the training, testing, and validation phases for the LSVM model predicting discharge disposition

**Fig. 3:**
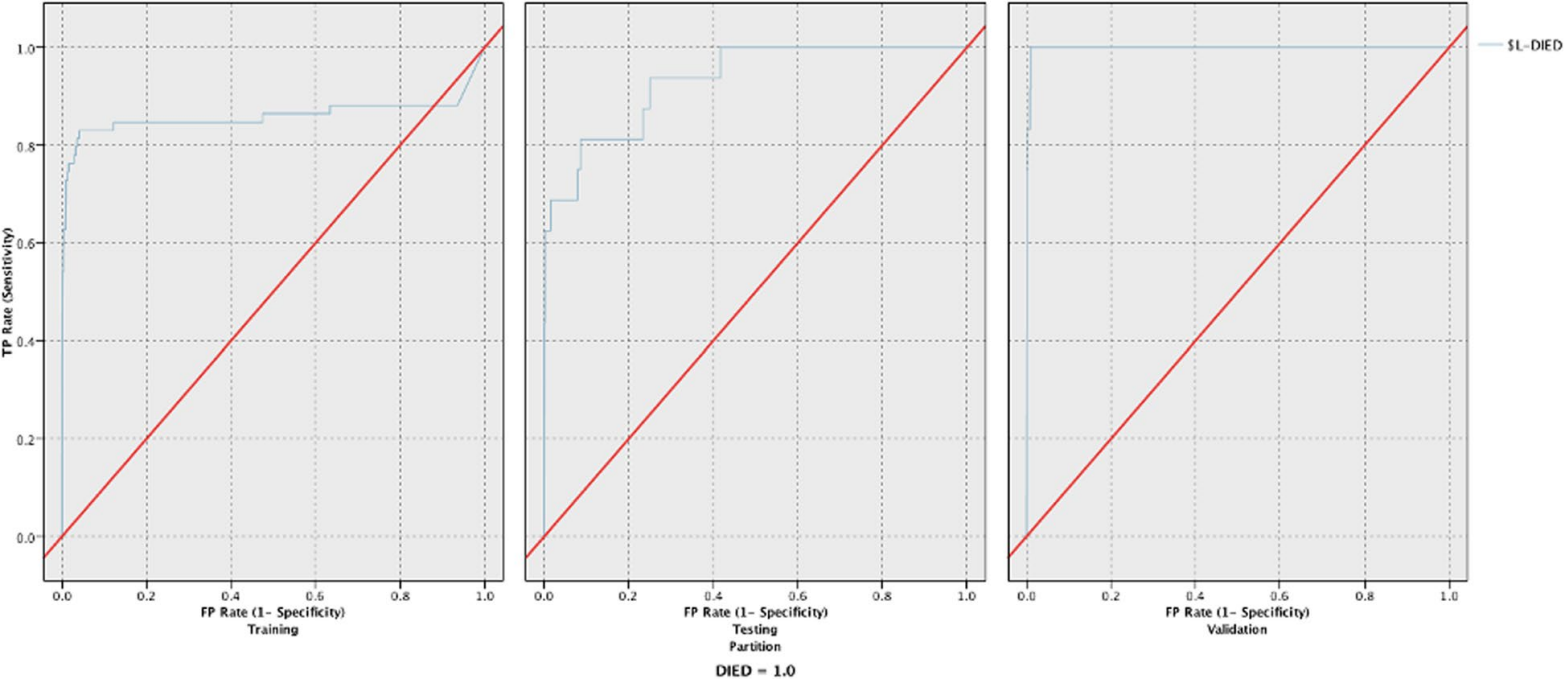
ROC curves for the training, testing, and validation phases for the LSVM model predicting in-hospital mortality

For models developed using all 15 variables, the three most responsive models for LOS were LSVM, Neural Network, and Bayesian Network, with poor results measuring 0.684, 0.668 and 0.664, respectively (Table 3). The three most reliable models for LOS were Decision List, LSVM, and CHAID. Decision List had a good reliability of 85.44%, while LSVM and CHAID had a poor reliability of 66.55% and 65.63%, respectively. Figure 1 provides the ROC curves for the training, testing, and validation phases for the LSVM model predicting LOS. The three most responsive models for discharge disposition were LSVM, XGT Boost Tree, and XGT Boost Linear had fair performance with respective values of 0.747, 0.747, and 0.722 (Table 4). The two most reliable models for discharge yielding good reliability were Decision List and LSVM measuring 89.81% and 80.26% respectively, and the third most reliable one for discharge with fair results was CHAID at 79.80%. Figure 2 provides the ROC curves for the training, testing, and validation phases for the LSVM model predicting discharge disposition. The top 4 models that yielded excellent responsiveness for in-hospital mortality were LSVM, XGT Boost Linear, Neural Network, and Logistic Regression. with their values being 0.997, 0.997, and 0.996, respectively (Table 5). The most reliable models, all with excellent reliability, for in-hospital mortality were XGT Boost Tree, Decision List, LSVM, and CHAID, with values of 99.98%, 99.91%, 99.89%, and 99.89%, respectively. Figure 3 provides the ROC curves for the training, testing, and validation phases for the LSVM model predicting in-hospital mortality.

**Table 3:** Responsiveness and reliability in predicting length of stay for the 10 models developed using all 15 variables

LOS						
	Reliability (Accuracy)			Responsiveness (AUC)		
	Training	Testing	Validation	Training	Testing	Validation
Random Forest	91.44%	60.86%	61.30%	0.94	0.632	0.636
Neural Network	62.81%	62.84%	62.79%	0.662	0.661	0.668
XGT Boost Tree	61.44%	61.40%	61.44%	0.619	0.615	0.61
XGT Boost linear	61.44%	61.40%	61.44%	0.603	0.6	0.595
LSVM	66.64%	66.84%	66.55%	0.689	0.689	0.684
CHAID	65.54%	65.41%	65.63%	0.665	0.665	0.663
Decision List	85.57%	85.39%	85.44%	0.59	0.593	0.59
Discriminant	59.29%	59.55%	59.12%	0.616	0.622	0.615
Logistic Regression	62.84%	62.87%	62.79%	0.662	0.662	0.661
Bayesian Network	62.99%	63.22%	63.03%	0.664	0.665	0.664

**Table 4:** Responsiveness and reliability in predicting discharge disposition for the 10 models developed using all 15 variables

Discharge						
	Reliability (Accuracy)			Responsiveness (AUC)		
	Training	Testing	Validation	Training	Testing	Validation
Random Forest	91.50%	74.25%	74.05%	0.955	0.671	0.675
Neural Network	75.62%	75.70%	75.53%	0.72	0.715	0.721
XGT Boost Tree	79.81%	79.81%	79.53%	0.749	0.741	0.747
XGT Boost linear	79.81%	79.81%	79.53%	0.719	0.715	0.722
LSVM	80.43%	80.43%	80.26%	0.745	0.742	0.747
CHAID	80.04%	80.02%	79.80%	0.712	0.711	0.713
Decision List	89.97%	90.03%	89.81%	0.648	0.647	0.648
Discriminant	64.49%	64.28%	64.35%	0.693	0.694	0.694
Logistic Regression	75.50%	75.63%	75.44%	0.716	0.713	0.718
Bayesian Network	75.14%	75.46%	75.13%	0.713	0.71	0.715

**Table 5:** Responsiveness and reliability in predicting mortality for the 10 models developed using all 15 variables

TKA Mortality						
	Reliability (Accuracy)			Responsiveness (AUC)		
	Training	Testing	Validation	Training	Testing	Validation
Random Forest	93.49%	93.49%	93.47%	0.941	0.687	0.749
Neural Network	93.47%	93.49%	93.47%	0.816	0.938	0.996
XGT Boost Tree	99.97%	99.97%	99.98%	0.921	0.839	0.954
XGT Boost linear	99.97%	99.97%	99.81%	0.982	0.938	0.997
LSVM	99.87%	99.89%	99.89%	0.981	0.944	0.997
CHAID	99.87%	99.89%	99.89%	0.978	0.901	0.991
Decision List	99.90%	99.90%	99.91%	0.845	0.925	0.871
Discriminant	86.61%	86.72%	86.39%	0.894	0.97	0.93
Logistic Regression	93.21%	93.26%	93.17%	0.86	0.932	0.996
Bayesian Network	93.47%	93.49%	93.47%	0.931	0.821	0.632

Separate models were then developed using the three most reliable algorithms for each outcome and their predictive performance was compared using either patient-specific or situational variables. Tables 6 and 7 describe the performance of models developed with patient-specific variables and situational variables, respectively. For nearly all outcomes, responsiveness was higher for each algorithm when trained with patient-specific variables vs. situational variables, the only exception being CHAID having marginally better performance for predicting LOS when developed with situational variables. Similarly, reliability was higher for most algorithms when models were developed using patient-specific as opposed to situational variables, with the exception of higher reliability for CHAID for predicting LOS and Decision List for predicting discharge disposition when developed using situational variables, and equivalent reliability of XGT Boost Tree and LSVM for predicting mortality when developed using either patient-specific or situational variables.

**Table 6:** Responsiveness and reliability in predicting length of stay, discharge disposition, and mortality for the best performing three models when trained with patient-specific variables only

	**Reliability (Accuracy)**			**Responsiveness (AUC)**		
	Training	Testing	Validation	Training	Testing	Validation
**LOS**						
LSVM	64.19%	64.11%	63.98%	0.646	0.649	0.642
CHAID	63.63%	63.83%	63.62%	0.634	0.634	0.63
Decision List	85.64%	85.50%	85.45%	0.586	0.59	0.586
**Discharge**						
LSVM	80.03%	80.07%	79.83%	0.721	0.721	0.723
CHAID	80.04%	80.02%	79.80%	0.706	0.707	0.708
Decision List	89.88%	89.87%	89.59%	0.648	0.649	0.651
**Mortality**						
XGT Boost Tree	99.87%	99.89%	99.89%	0.851	0.883	0.888
LSVM	99.87%	99.89%	99.89%	0.907	0.951	0.941
Decision List	99.90%	99.90%	99.91%	0.845	0.925	0.871

**Table 7:** Responsiveness and reliability in predicting length of stay, discharge disposition, and mortality for the best performing three models when trained with situational variables only

	**Reliability (Accuracy)**			**Responsiveness (AUC)**		
	Training	Testing	Validation	Training	Testing	Validation
**LOS**						
LSVM	62.07%	61.78%	62.04%	0.587	0.586	0.584
CHAID	64.92%	64.88%	65.10%	0.648	0.651	0.649
Decision List	84.45%	84.61%	84.26%	0.559	0.556	0.561
**Discharge**						
LSVM	79.81%	79.81%	79.53%	0.581	0.581	0.581
CHAID	79.81%	79.81%	79.53%	0.585	0.582	0.583
Decision List	90.97%	91.05%	91%	0.557	0.553	0.554
**Mortality**						
XGT Boost Tree	99.87%	99.89%	99.89%	0.5	0.5	0.5
LSVM	99.87%	99.89%	99.89%	0.639	0.494	0.514
Decision List	58.31%	58.29%	58.53%	0.571	0.455	0.536

## Discussion

TKA is one of the most common procedures performed in the United States, with a considerable associated economic burden. As healthcare systems continue to aim to optimize value of care delivery, there has been a growing focus on standardizing outcomes and establishing accurate risk assessment prior to TKA [5, 19]. More recently, ML has been applied to develop models to predict outcomes after TKA [8, 13, 14, 20]. As such, the aim of this study was to develop and compare the performance of multiple ML models to predict in-hospital mortality, LOS, and discharge disposition after TKA and to compare the performance of models trained using patient-specific and situational variables.

Selecting an appropriate algorithm for training is critical in developing a predictive ML model. As the number of ML algorithms abounds, there has been a concerted effort within the medical literature to compare ML algorithms to identify which are optimal for a given set of data and diseases [21, 22]. However, within the nascent orthopedic ML literature, different ML algorithms have been seldom compared when developing predictive models. Therefore, this study aimed to assess the performance of ten different ML models for prediction of LOS, mortality, and discharge disposition after TKA. When comparing the different ML models using fifteen independent variables available in the NIS database, the LSVM methodology was consistently the most responsive and reliable one, being within the top three best-performing ML models in predicting all tested outcomes. This result is not surprising, as support vector machine algorithms have consistently been one of the most widely used ML predictive algorithms [22]. Still, other studies in the general medical literature have shown superior performance of other algorithms for the prediction of other outcomes [21–23]. As such, it should be noted that if different variables or outcomes are to be tested in a different study, it is possible that a different ML algorithm would be more effective and accurate within its predictive capacity. As clinical application of ML continues to evolve, it should be stressed that various ML methodologies should be tested prior to developing and deploying a model for clinical use.

The selection of the optimal independent variables or features to train models is a cornerstone of supervised ML. Redundant variables can complicate models without increasing the predictive accuracy, while a deficiency of variables can oversimplify models without capturing the true complexity of a given use case. In the nascent TKA-related ML literature, there has typically been little justification for the variables selected to train models. Therefore, the predictive capacity of various models trained with either patient-specific or situational variables were compared. As both patient-specific factors, such as age, and situational variables, such as hospital volume, have been shown to correlate with outcomes after TKA, this distinction would be useful for the development of future models for use in clinical practice. Our analysis demonstrated consistently better performance of models developed with the 8 patient-specific variables when compared to models developed using 7 situational variables. These results, while stressing the importance of patient-specific variables, also highlight the potential of a smaller number of variables to develop equivalent predictive models. A similar concept was demonstrated recently in a study on heart failure patients that reported equivalent performance of an ML model using only 8 variables compared to one using a full set of 47 variables [24]. Continued research within the orthopedic literature on variable engineering and selection is critical, and identifying the most predictive variables will prove useful for the development of models that will be deployed to clinical practice.

There were several limitations to this study. The strength of ML models is dependent on the quality of the data used to train, test, and validate the algorithms, and administrative databases may be prone to incompleteness and errors [25]. However, the NIS has been demonstrated as an appropriate database to utilize for predictive large population-based studies and administratively-coded comorbidity data has been previously validated as accurate [26]. Another limitation is that the LOS outcome was adjusted to be binary to simplify outcomes and provide more accurate analysis. These adjusted outcomes are useful in the setting of predictive ML at the expense of precise predictions. However, despite the continuous nature of LOS as a variable, when quality-improvement efforts are implemented in the clinical setting, the target for improvement in LOS is generally a binary cutoff, and so a binary predictive model has practical use. Another limitation is that the findings of this study were not externally validated. Although external validation was not within the scope of the study, efforts were made to internally validate the results, as the dataset was split into 64% training, 16% testing, and 20% validating groups. The analysis of each phase was concurrent with all models with similar results, indicating the internal validity of the findings. Still, comparison with another data source would be useful to assess the generalizability of each ML model and the replicability of the findings in this study.

There were several strengths to this study. This study represents a novel attempt in the orthopedic literature to analyze a large variety of ML algorithms to develop the best-performing model. Our analysis of multiple ML algorithms generates insights into the performance of these various algorithms for multiple outcomes, which has seldom been encountered in the orthopedic literature. Additionally, by demonstrating the generally superior performance of models trained on patient-specific variables over situational variables, this study highlights the role that patient-specific factors play in determining critical quality outcome metrics within the available dataset. These insights should empower efforts aimed to influence both clinical practice and reimbursement models, which typically do not consider patient factors despite their demonstrably substantial impact on various quality metrics.

## Conclusion

In summary, this study compared ten ML models developed using different algorithms to predict three important quality metrics: mortality, LOS, and discharge disposition. Models developed using patient-specific variables performed better than models developed using situational variables. As the effort to develop ML models and identify which ML algorithms are optimal for a given set of conditions and outcomes, these results prove useful in the development of predictive ML models for accurate risk assessment and stratification for TKA.

## Supplementary Information

**Additional file 1.** ICD-10-PCS codes utilized to identify primary TKA recipients.

## Data Availability

The datasets generated and/or analyzed during the current study are available in the National Inpatient Sample repository, https://www.hcup-us.ahrq.gov/db/nation/nis/nisdbdocumentation.jsp.
