# Supplementary material for: Improved performance of machine learning models in predicting length of stay, discharge disposition, and inpatient mortality after total knee arthroplasty using patient-specific variables
Source: Arthroplasty. 2023 Jul 2;5:31. doi: 10.1186/s42836-023-00187-2 (PMC10315023; doi:10.1186/s42836-023-00187-2)
Supplement: Supplementary file 1 — Additional file 1. ICD-10-PCS codes utilized to identify primary TKA recipients. [file 42836_2023_187_MOESM1_ESM.docx]

Additional file 1

ICD-10-PCS codes utilized to identify primary TKA recipients

|  | Cemented | Uncemented |
| --- | --- | --- |
|  |  |  |
|  |  |  |
| Right TKA | 0SRC0J9 | 0SRD0JA |
| Left TKA | 0SRD0J9 | 0SRC0JA |
